# Clinical education alone is sufficient to increase resistance training exercise prescription

**DOI:** 10.1371/journal.pone.0212168

**Published:** 2019-02-27

**Authors:** Gavin Williams, Linda Denehy

**Affiliations:** 1 Epworth Hospital, Melbourne, Australia; 2 School of Physiotherapy, The University of Melbourne, Melbourne, Australia; 3 Melbourne School of Health Sciences, The University of Melbourne, Melbourne, Australia; Berner Fachhochschule, SWITZERLAND

## Abstract

A large body of evidence demonstrates that resistance training has been ineffective for improving walking outcomes in adults with neurological conditions. However, evidence suggests that previous studies have not aligned resistance exercise prescription to muscle function when walking. The main aim of this study was to determine whether a training seminar for clinicians could improve knowledge of gait and align resistance exercise prescription to the biomechanics of gait and muscle function for walking. A training seminar was conducted at 12 rehabilitation facilities with 178 clinicians. Current practice, knowledge and barriers to exercise were assessed by observation and questionnaire prior to and immediately after the seminar, and at three-month follow-up. Additionally, post-seminar support and mentoring was randomly provided to half of the rehabilitation facilities using a cluster randomised controlled trial (RCT) design. The seminar led to significant improvements in clinician knowledge of the biomechanics of gait and resistance training, the amount of ballistic (*t* = -2.38; *p* = .04) and conventional (*t* = -2.30; *p* = .04) resistance training being prescribed. However, ongoing post-seminar support and mentoring was not associated with any additional benefits *F*(1, 9) = .05, *p* = .83, partial eta squared = .01. Further, improved exercise prescription occurred in the absence of any change to perceived barriers. The training seminar led to significant improvements in the time spent in ballistic and conventional resistance training. There was no further benefit obtained from the additional post-seminar support. The seminar led to improved knowledge and significantly greater time spent prescribing task-specific resistance exercises.

## Introduction

The primary cause of reduced ability to walk for many people with neurological conditions is muscle weakness [1, 2]. In the field of stroke rehabilitation, where evidence is most advanced, clinical guidelines state a ‘strong’ recommendation for resistance training for the lower limb to improve walking [3, 4]. However, despite the strong recommendation, the guidelines state ‘the optimal strengthening protocol is not known’ [3]. Despite a large body of evidence that muscle weakness is the primary impairment causing walking limitations, and guidelines recommending resistance training during rehabilitation, systematic reviews demonstrate that resistance training has little impact on walking outcomes [5, 6]. Further, observational studies have identified that clinicians are devoting a reasonable proportion of time to resistance training during rehabilitation [7], but the exercises prescribed may not necessarily be specific to the goal of walking [8, 9]. Task-specific resistance training is a term used to describe exercises specifically prescribed to replicate muscle performance for function, with the expectation that resistance gains will more likely lead to improved function, in this case walking. With respect to walking, a key aspect of task-specific resistance training is the speed at which exercises are performed. The main lower-limb joints move quickly at high angular velocities during walking [10], indicating that resistance exercises should be performed ballistically [11]. Ballistic, or fast, resistance exercises are prescribed to improve power generation [11]. ‘Power’ is a term that describes how quickly force is generated during a movement or exercise, and is important for walking when the joints are required to move quickly [12]. It is possible that significant improvements in leg resistance have not translated to greater walking outcomes because the resistance exercises prescribed have not been specific to training the muscles responsible for power production during walking [9], guidelines for specificity of resistance training are not commonly followed [8], and resistance exercises have been prescribed at slow speeds [9].

The limited impact of guideline adherence on mobility outcomes may relate to the guidelines themselves. The AGREE II statement is a 23-item tool that outlines guideline development, reporting and evaluation [13]. It states what is required of a guideline for clinical implementation. In the case of the Stroke Foundation guidelines [3], the recommendation for resistance training programs, especially for the lower extremity, is not supported by any further tools or advice on how to implement resistance training. This means that clinicians who do follow clinical guidelines are not provided with instructions or strategies for how to perform resistance training, which exercises they should be performing, how they should be progressed or how often they should be performed.

The biomechanics of, and muscle function for, walking are well established [14–18]. It is possible that the knowledge of biomechanics of gait and muscle function during walking, coupled with the application of resistance training guidelines in people with lower limb muscle weakness have not been readily applied in adult neurological rehabilitation [9]. To improve mobility outcomes for adults with neurological conditions, a range of therapist-related implementation factors may need to be considered [19]. If clinicians are devoting a reasonable proportion of time to resistance training during rehabilitation [7], then education regarding the application of task-specific resistance exercises may be sufficient to improve patient outcomes. However, implementing change in clinician behaviour, or in this case resistance exercise prescription, is complex and multi-factorial [19] and there is wide-spread agreement amongst researchers that education alone is not usually sufficient to lead to change in clinical practice.

There are a number of task-specific criteria outlined by the American College of Sports Medicine which are important to consider when prescribing resistance exercises. They relate to factors such as appropriate targeting, duration, and load etc [11]. However, the overall aim of this project was to determine the effectiveness of a seminar (including education, demonstration and practical application) on improving the proportion of ballistic resistance exercise prescription for treating muscle weakness in people with neurological conditions who were receiving therapy to improve their ability to walk. Specifically, the four main aims were to;

- Determine whether attendance at a training seminar resulted in changes in ballistic resistance exercise prescription by treating therapists for people with mobility limitations,
- Evaluate the effectiveness of a training seminar for improving knowledge of the biomechanics of gait, and task-specific (ballistic) resistance training exercises for walking,
- Identify barriers and enablers to change in resistance exercise prescribing patterns,
- Determine whether ongoing support and mentoring was associated with higher levels of change in exercise prescription.

## Methods

This project was approved by the Human Research and Ethics Committee of The University of Melbourne (Ethics ID: 1647064). The individuals pictured in the video summary of the training seminar provided written informed consent (as outlined in PLOS consent form) to publish their image alongside the manuscript. A training seminar was developed to improve the knowledge and exercise prescription of clinicians working with people with neurological conditions who had leg weakness and walking limitations. The training seminar was piloted at four rehabilitation facilities prior to the commencement of this project. Feedback was incorporated into the final version of the program, and delivered by the primary author.

The seminar was conducted at 12 separate rehabilitation facilities comprising 3.5 hours of education, training, practical demonstration and modelling, and participant practice. The education content included information regarding current guidelines for muscle weakness in neurological conditions, the evidence contributing to the development of these guidelines, the evidence for resistance training in adult neurological rehabilitation, the biomechanics of walking, muscle function for walking, the American College of Sports Medicine (ACSM) guidelines for task-specific (ballistic) resistance training [11], case presentations, modelling and demonstration of exercise prescription, and clinician practice of exercise application. The seminar utilised a lecture format, practical demonstration and clinician practice. A video recording of the training seminar is available by contacting the lead author. All clinicians (physiotherapists and exercise physiologists) at each rehabilitation facility were approached prior to the seminar and provided with written plain language statements. Those who agreed to participate in this project provided written informed consent. Attendance was free and voluntary. Signed informed consent forms were collected separately from the questionnaires to ensure anonymity.

Questionnaires and direct observation at three timepoints were used to collect outcome data. Timepoint 1 was prior to the seminar, Timepoint 2 was immediately following the seminar and Timepoint 3 was three months after the seminar. Questionnaires were completed at all three timepoints. The questionnaires were used to collect participant demographic data and to evaluate 1) baseline knowledge (Timepoint 1); 2) knowledge gained during the seminar (Timepoint 2); and 3) how knowledge was retained (Timepoint 3). Bespoke questionnaires were designed by the authors given the novel nature of this study, and pilot tested on six clinicians working in neurological rehabilitation. Feedback from the clinicians, in relation to clarity, wording and meaning, was incorporated into the final versions provided to the clinicians who participated in this study. The direct observation sessions occurred at Timepoints 1 and 3 to evaluate change in exercise prescription. Each observation session was conducted over one full working day.

**Fig 1:**
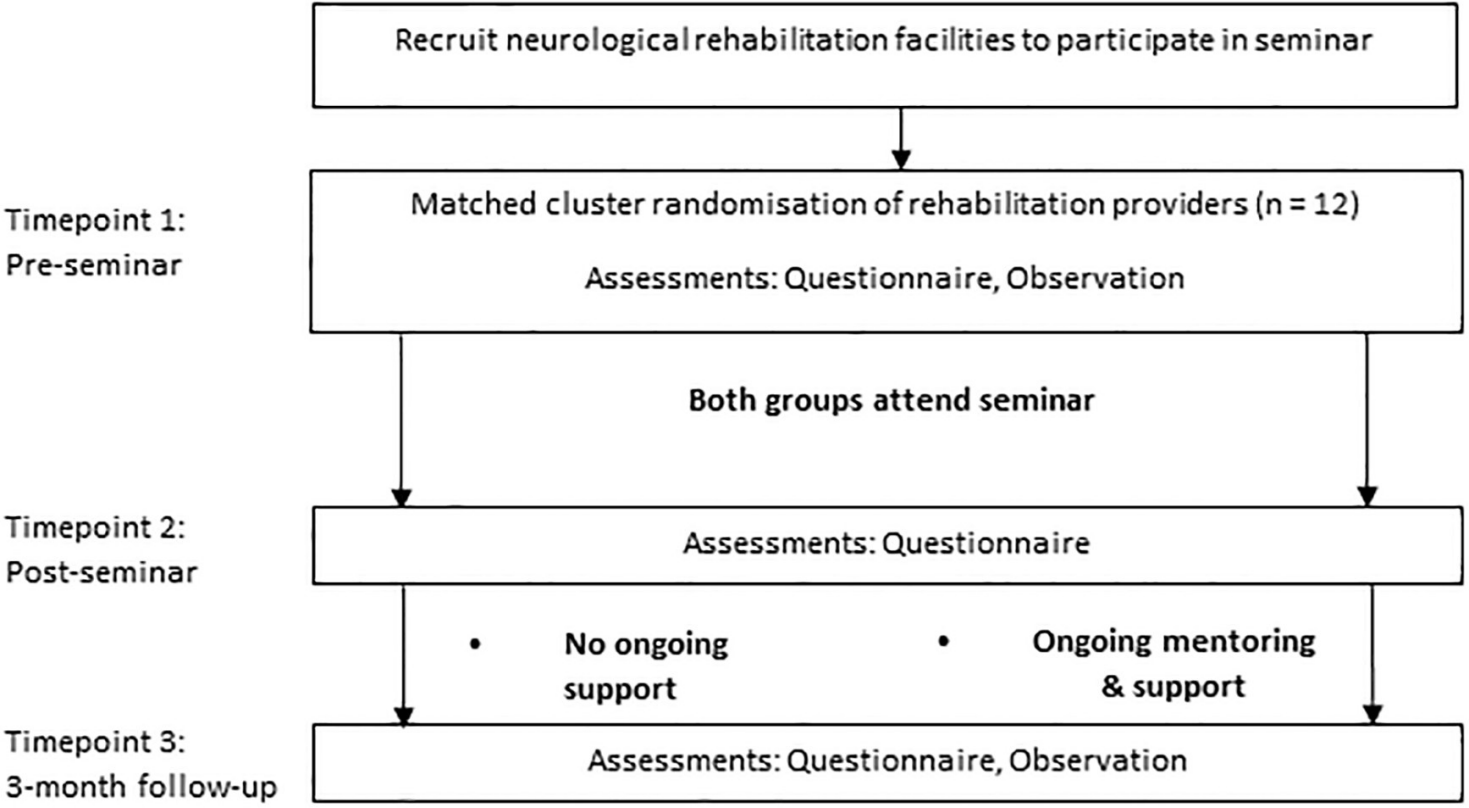
Study flow diagram.

Since the need for additional implementation and behaviour change strategies and support after the seminar was unclear, the rehabilitation facilities were randomised to either receive additional mentoring and support or none. A matched cluster design was piloted (Fig 1) to determine whether ongoing support and mentoring after the seminar was associated with higher levels of change in exercise prescription (Aim 4). Twelve rehabilitation facilities were identified, from a list of 14, and consented to participate in the observational stage of this project, representing the public and private sectors, and metropolitan and rural areas. The four public, four private and four rural rehabilitation facilities were each selected as they employed the largest number of therapists in their respective sector. Two facilities were not approached to participate due to resistance training trials that were concurrently being conducted. The four rehabilitation facilities within each cluster were randomised by an independent biostatistician, using consecutively-numbered opaque envelopes. Each rehabilitation facility was classified as public or private, and metropolitan or rural, matched and then randomly assigned to receive ongoing support and mentoring, or no support. The design of the matched cluster RCT conformed to the Consort 2010 statement: extension to cluster randomised trials [20]. However, as this was a pilot project, the trial was not registered.

### Intervention group

Half of the rehabilitation facilities were matched cluster randomised to receive ongoing mentoring and support for three months. The ongoing support was provided by the lead investigator. The lead investigator liaised with a locally nominated ‘change champion’ at the rehabilitation facility weekly for the first four weeks, and then fortnightly after that. Strategies were provided to address potential barriers identified in the pilot study, in previous rehabilitation studies, and any further barriers identified by the local change champion. Identification of barriers to change in professional behaviours is important as targeting strategies may be effective if barriers have been identified [21]. The potential barriers identified were grouped into six domains which included 1) leadership, 2) variations in staffing, 3) patient safety, 4) therapist beliefs about resistance training, 5) therapist confidence, motivation and engagement, and 6) resources (time and equipment). The specific strategies employed to facilitate uptake were;

- Designation of a local change champion at each site.
- Regular staff inservice education, journal clubs and practical sessions focusing on the content provided in the seminar were conducted facilitate engagement and engage new staff entering the rehabilitation casemix.
- Change champion led practical sessions were conducted so staff could practice implementing new exercises and address any safety concerns.
- Further educational material was provided to the rehabilitation facilities to support therapist understanding, beliefs and engagement, primarily in the form of research articles and targeted text-books.
- Discussion of issues related to implementation were raised at staff team meetings lead by the change champion, in addition to the inservice education program, to ensure ongoing engagement.
- Practical demonstrations using existing equipment and resources.

The change champion at each of the six sites was responsible for what type, and how much of each strategy was used to facilitate implementation of ballistic exercises into the usual care of people with neurologically related mobility limitations. The type and frequency of specific strategies employed to facilitate uptake was not prescribed or controlled. Utilisation of the strategies and information provided was at the discretion of the local change champion at each site.

### Control group

Participants (therapists) in the control group attended the seminar, and completed the questionnaires at all three timepoints, but did not receive any further support following the seminar.

### Primary outcome measure

The primary outcome measure for this project was the amount of time (minutes) spent performing task-specific (ballistic) resistance training provided to improve walking outcomes. An independent and blinded observer collected the observational data during one full clinical day (typically 9am– 4pm with an hour break) in each rehabilitation facility prior to, and three-months after the seminar. Data related to the number of neurological clients with walking limitations, and the type of exercises performed were recorded on a specific data collection sheet (S1 File).

### Secondary outcome measures

The secondary outcome measures related to knowledge of the biomechanics of walking, muscle function during walking, and the principles of resistance training. Knowledge was assessed by questionnaires completed prior (Timepoint 1), immediately after (Timepoint 2) and three-months following (Timepoint 3) the seminar (S2–S4 Files). Correct responses on the questions related to knowledge were used for comparison. The protocol for this study is available at dx.doi.org/10.17504/protocols.io.wsyfefw.

### Data analysis

Descriptive statistics were used to summarise the demographic data.

To address the four aims, the following analyses were performed;

1. To determine whether attendance at the seminar resulted in changes in resistance exercise prescription, a paired samples *t* test was performed between the observational data collected at timepoints 1 and 3.
2. To evaluate the effectiveness of the seminar, comparison of questionnaire results from timepoints 1 and 2. A further comparison was conducted between timepoints 1 and 3 to evaluate retention of knowledge gained from the seminar. Analyses were conducted using a Wilcoxon Signed Rank Test or Chi-squared test. Effect sizes for the Chi-squared test were calculated using the phi coefficient, using Cohen’s criteria of .10 for a small effect, .30 for a medium effect and .50 for a large effect [22].
3. Descriptive statistics were used to summarise the rates at which therapists identified barriers and enablers to change in resistance exercise prescribing patterns.
4. To determine whether ongoing support and mentoring was associated with higher levels of change in exercise prescription, a one-way between groups analysis of covariance (ANCOVA) was performed.

## Results and discussion

### Therapists

Table 1 summarises the number of therapists that completed the questionnaires at each timepoint, and the number of patients that were observed. An average of 14.8 clinicians (range 8–32: IQR 9–17) attended each seminar. Table 1 demonstrates that there was a similar representation from each of the public, private and rural centres. At three-month follow-up, 80% of the respondents who completed the post-seminar questionnaire (timepoint 2) returned the final questionnaire (timepoint 3). The majority of therapists who participated were physiotherapists (n = 158; 88.8%) and female (n = 151; 84.8%). Therapist experience was evenly distributed across the categories; < 2 years n = 49 (27.5%), 3–5 years n = 46 (25.8%), 6–10 years n = 36 (20.2%), and > 10 years’ experience n = 47 (26.4%).

**Table 1:** Number of therapist questionnaires and patient observation sessions completed.

	Questionnaire timepoint			%^a^	Observation session	
	1	2	3		1	2
**Entire cohort**	178	170	136	80.0	68	81
**Public facilities**	60	60	46	79.9	17	26
**Private facilities**	56	53	42	80.7	26	25
**Rural facilities**	62	57	48	87.3	25	30

^a^indicates the proportion of those who having completed questionnaire 2, also completed questionnaire 3.

### Results Aim 1: The impact of the seminar on exercise prescription

Table 1 shows that there were 68 patients with neurologically related gait disorders observed across the 12 sites initially, and a further 81 patients observed three months later. The greater number of patients observed during the second session was unintended, and simply reflects that more patients with mobility limitations attended on those particular days. Overall, there were only 30.5 minutes of ballistic exercise initially performed by the 68 patients from a total 2188 minutes (1.4%) spent exercising. Three months later, 122 minutes (4 times greater) of ballistic exercise was performed by the 81 patients during 3288 minutes (3.7%) spent exercising. Compared to ballistic resistance training, a greater amount of conventional resistance training was initially performed, totalling 200 minutes (9.1%). Time spent performing conventional resistance training also increased at follow-up to 340 minutes (10.3%). At each rehabilitation facility, the average time spent performing ballistic exercise per site was only 2.5 (±5.4) min initially. The amount of time spent performing ballistic exercise increased significantly to 10.2 (±9.7) min at follow up (*t* = -2.38; *p* = .04). The eta squared statistic (0.08) indicates a moderate effect size [22]. Average time spent engaged in conventional exercise also increased significantly from 16.7 (±14.4) min initially to 28.3 (±22.7) min at follow-up (*t* = -2.30; *p* = .04). The eta squared statistic (0.07) indicates a moderate effect size.

### Results Aim 2: The impact of the seminar on knowledge

Overall, there were large improvements in the proportion of participants that reported improved confidence in their knowledge (Table 2) and correctly answered the knowledge-based questions at timepoints 2 & 3 (Table 3). Table 3 summarises the number and proportion of correct responses to the biomechanics questions at baseline, after the seminar and at three-month follow-up.

**Table 2:** Clinician self-reported confidence in their knowledge prior to and after the seminar.

	Timepoint 1		Timepoint 2	
	Strongly agree	Agree	Strongly agree	Agree
Biomechanics of walking	6.2%	59.3%	47.1% ^a^	52.9%
Muscle function during walking	5.9%	52.0%	52.9% ^a^	46.5%
Impact of the UMNS	6.8%	56.5%	28.8% ^a^	64.1%
Principles of resistance training	6.8%	63.8%	55.3% ^a^	43.5%

^a^ *p* < .001 (Wilcoxon Signed Rank Test)

UMNS: Upper Motor Neurone Syndrome

**Table 3:** Number and proportion of correct responses to the biomechanics of gait and principles of resistance training questions.

Question	Timepoint 1 n (%)	Timepoint 2 n (%) ES (*r*)		Timepoint 3 n (%) ES (*r*)	
Three most important muscle groups for forward propulsion when walking	35 (20.2)	151 (88.8) ^a^	0.51	96 (70.6) ^a^	0.39
Primary role of the quadriceps	71 (41.0)	140 (82.3)		91 (66.9)	
Primary role of the hamstrings	91 (52.6)	118 (69.4) ^b^	0.22	99 (72.8)	
Active phase of the hip extensors	25 (14.5)	121 (71.2)		71 (52.2)	
Active phase of the ankle plantarflexors	127 (75.1)	150 (88.2)		121 (89.0)	
Main strategy to increase walking speed	36 (21.1)	135 (79.4)		69 (50.7)	
Contribution of the ankle joint to overall leg power generation	59 (34.3)	124 (72.9)		78 (57.4) ^b^	0.27
Contribution of the Achilles tendon to ankle power generation	14 (8.2)	136 (80.0)		79 (58.1)	
Ipsilateral compensation strategy	12 (7.2)	40 (23.5) ^a^	0.14	20 (14.7)	
Roles of the five main muscle groups	27 (16.2)	97 (57.1) ^a^	0.35	55 (40.4) ^a^	0.22
ACSM guidelines for specificity	76 (46.6)	108 (63.5)		71 (52.2)	
Main strategies resistance training progression	52 (31.7)	89 (52.4)		72 (52.9)	
Power is measured as rate of force production	67 (41.4)	117 (86.0)		93 (68.4)	

ES = effect size. Effect sizes reported for Chi-squared test are the phi coefficient.

^a^ Significant difference (*p* < .01) compared to Timepoint 1 on a Wilcoxon Signed Rank Test

^b^ Significant difference (*p* < .01) compared to Timepoint 1 on a Chi-squared test for independence

### Results Aim 3: Barriers and enablers

When comparing the perceived barriers to implementing ballistic resistance training after the seminar and at three-month follow-up, there was little change. The same three barriers were most commonly reported by therapists (Table 4). They were 1) their perception of the patients’ capacity to engage in ballistic resistance training, 2) the therapists’ confidence and capacity to provide ballistic resistance training to patients and 3) access to equipment. Although the barriers did not change, therapists did engage in more ballistic and resistance training, and reported they were more confident to do so.

**Table 4:** Clinician reported barriers and enablers (number and proportion).

	Timepoint 1 n (%)	Timepoint 2 n (%)
Barriers		
Patient capacity	113 (63.8)	96 (70.6)
Therapist confidence	91 (51.4)	53 (39.0)
Equipment	89 (50.3)	71 (52.2)
Enablers		
Knowledge of biomechanics		99 (72.8)
Physical impairments		96 (70.6)
Improved confidence		83 (61.0)
Resistance training principles		64 (47.1)
Importance of ballistic training		51 (37.5)

In relation to enablers, therapists reported at three-month follow-up that the most important factors were related to knowledge (Table 4). They included 1) a greater understanding of the primary muscle groups responsible for forward propulsion when walking, 2) a better understanding of the contribution of physical impairments to gait disorders, 3) improved confidence and capacity to provide ballistic resistance training to patients, 4) a greater understanding of resistance training principles and application of exercise programs, and 5) a belief that targeted ballistic resistance training is the best intervention for their patients.

### Results Aim 4: The impact of ongoing support and mentoring

A one-way between groups ANCOVA was conducted to compare the effectiveness of the ongoing support and mentoring. After adjusting for pre-seminar ballistic resistance training time, there was no significant difference between the two groups following the seminars, *F*(1, 9) = .05, *p* = .83, partial eta squared = .01.

## Discussion

The training seminar led to significant improvements in the time spent in ballistic and conventional resistance training. Further, improved exercise prescription occurred in the absence of any change to perceived barriers. However, there was no additional benefit associated with ongoing mentoring and support.

The results indicate that the primary barrier to therapists prescribing more ballistic resistance exercises was knowledge. As knowledge improved, so too did the amount of ballistic and conventional resistance training prescribed. Although the improvements in task-specific resistance training were significant, they were obtained from a low baseline, and were small in magnitude. This finding is consistent with a recent Cochrane review that reported tailored implementation can be effective but effects tend to be small to moderate [21]. Large improvements in clinician knowledge were obtained, demonstrated by the number of clinicians responding correctly (Table 3). Improved exercise prescription occurring in the absence of any change to perceived barriers may be due to the inclusion of strategies for implementation, clinical examples, clinician practical application, and discussion of approaches to address potential barriers and utilize facilitators to exercise prescription. The inclusion of these additional tailored strategies, generated from the earlier pilot study, to the traditional lecture style format of post-graduate education may also be the reason why significant increases in ballistic exercise prescription were observed [21]. Reasons for the finding of no additional benefit associated with the ongoing mentoring and support may be that the seminar content was sufficient for this initial stage of implementation [19]. Alternatively, ongoing support may be required over a longer time period than that measured in this study to embed changes in work practices [19].

Improvements in the provision of ballistic resistance training were also obtained even though the barriers reported by therapists were unchanged. Prior to, and following the seminar the three mostly commonly reported barriers were beliefs about patient capacity to perform ballistic exercises, therapist confidence in delivering them, and access to appropriate equipment. Three months is a short period of time to change therapist confidence and their beliefs about patient capacity to engage in new exercises, when nearly half of the clinicians had more than five years of experience. Although confidence was a commonly reported barrier initially, it had improved by three-month follow-up, and was also one of the most commonly reported enablers. This indicates that therapists are in the early stages of behaviour change, as they begin to implement new exercises but are yet to establish a skill for a novel application [23–25].

A greater proportion of therapist time was spent performing conventional rather than ballistic resistance training before and after the seminar. This finding is in line with ACSM guidelines which suggest that a base of resistance training is required prior to ballistic resistance training [11]. Ballistic resistance training is novel to neurological rehabilitation, with few studies reporting its application in people with neurological conditions [8, 26–28]. Consequently, clinicians may remain cautious with regard to its application. Nevertheless, the ballistic application of exercise prescription was highlighted in this seminar due to the task-specificity of resistance training required to improve walking and was effective at linking established knowledge bases and facilitating some clinicians to implement these novel ballistic resistance exercises. Clinicians reported greater knowledge of the principles of resistance training, biomechanics of walking and muscle function, and increased their patients time spent performing resistance exercises including ballistic exercises. Not all lower-limb resistance and mobility related goals, such as sit to stand or transfers, require such fast or ballistic contractions. Overall, the significantly greater time spent performing ballistic and conventional resistance training indicates a greater appreciation of muscle weakness as the physical impairment for many people with neurologically related walking limitations.

### Limitations

The significant increase in ballistic resistance training is encouraging, but that does not necessarily indicate that all the ballistic exercises prescribed were task-specific. Speed of muscle contraction is only one aspect of task specificity. For a resistance exercise to be task specific, other factors such as type of contraction, segmental alignment, and load need to be considered [11], and evidence at this stage suggests task specificity of resistance exercises in neurological rehabilitation is poor [8]. The impact of task-specific lower limb leg resistance on walking outcomes is yet to be reported.

The seminars led to a significant increase in total time spent engaged in ballistic and conventional resistance training, and improved therapist knowledge and confidence, but care must be taken in interpreting these results. There was an increase in the number of people observed at Timepoint 2, and therefore the total number of minutes spend exercising, which may have influenced the results. However, in relation to resistance training, the proportion of time spent on ballistic and conventional resistance exercise increased at Timepoint 2. Further, it is unknown whether the patients benefitted. It is possible that the response options in the post-seminar questionnaire may have biased results towards more positive answers as several of the questions were not neutrally formatted (for example ‘Do you have a better understanding’). The questions were specifically worded this way to determine whether participants perceived that the training seminar had an effect on their knowledge, and although the questions are not neutrally formatted, the response categories were. Further research needs to determine whether the application of task-specific ballistic resistance training for walking is more effective than conventional resistance training. If task-specific ballistic resistance training is more effective than current resistance training practices, then the Stroke Foundation (and other) guidelines can be further developed [3], and include advice and tools for clinical implementation.

The improved rates of conventional and ballistic resistance training three-months following the seminar may be due to an observer or Hawthorne effect. Clinicians were unaware of the purpose of the initial observation sessions, other than a general quantification of the types of exercises routinely performed in physiotherapy. However, at three-month follow-up, clinicians would have had a greater awareness of the purpose of the observation session. We are unable to determine whether the amount of time spent performing ballistic resistance training during the observation sessions is an accurate reflection of usual practice, or partly due to an observer effect.

Finally, although post-seminar support was provided to half of the rehabilitation facilities, this support was not prescribed, controlled or measured. We took this pragmatic approach so that each site’s change champion was able to tailor the ongoing support to their facility’s needs. We found no difference in the amount of ballistic exercise prescribed between the sites randomised to receive post-seminar support, and those who received none, but we are unable to determine whether greater amounts of post-seminar support, or types of support, were associated with greater ballistic resistance training prescription at each site.

## Conclusion

The seminar led to improved knowledge and significantly greater time spent prescribing ballistic and conventional resistance training. Whilst significant, these improvements were small and achieved from a low baseline. There was no further benefit obtained at the sites randomised to receive additional post-seminar support. However, the seminar did include strategies for implementation, and addressed potential barriers and facilitators to exercise prescription.

## Supporting information

S1 File Observation checklist. (PDF)

S2 File Pre-seminar questionnaire. (PDF)

S3 File Post-seminar questionnaire. (PDF)

S4 File 3-month follow-up questionnaire. (PDF)

S5 File Study protocol. (PDF)

S6 File Clinical education alone is sufficient to increase resistance training exercise prescription summary. (MP4)
