# Supplementary material for: Clinical education alone is sufficient to increase resistance training exercise prescription
Source: PLoS One. 2019 Feb 27;14(2):e0212168. doi: 10.1371/journal.pone.0212168 (PMC6392279; doi:10.1371/journal.pone.0212168)
Supplement: S2 File — (PDF) [file pone.0212168.s002.pdf]

Questions about you

Professional background:
- ☐ Physiotherapist
- ☐ Exercise Physiologist
- ☐ Exercise Scientist

Years of experience since graduation:
- ☐ < 2 years
- ☐ 3-5 years
- ☐ 6-10 years
- ☐ >10 years

Normal work hours:
- ☐ < 10 hours/week
- ☐ 10-25 hours/week
- ☐ 25-40 hours/week
- ☐ >40 hours/week

Main work setting (tick all that apply):

☐ Inpatients	☐ Outpatients	☐ Community
☐ Acute	☐ Rehabilitation	☐ Private practice
☐ City	☐ Major regional facility	☐ Rural/remote
☐ Public Hospital	☐ Private Hospital	

☐ Other…………………………………………………….

Have you attended a post-graduate educational lecture/workshop/training session on the management of gait disorders for people with neurological conditions in the past 5 years?

- ☐ Yes
- ☐ No

In relation to gait disorders and gait training, where do you most commonly seek up to date knowledge and information? Please order these from 1-8 (1 – most common, 8 – least common)

- ☐ International guidelines
- ☐ Professional development activity (internal – i.e. provided by your employer)
- ☐ Professional development activity (external)
- ☐ Workshops/Conferences/seminars
- ☐ Books or Journal articles
- ☐ Online Resources
- ☐ Unit of university course
- ☐ Other (please list)…………………………………………………………

Questions about the type of patients you treat

Nominate the patient population you most commonly treat (i.e. 1-11 with '1' being the most common and '10' being the least common):

- ☐ Stroke
- ☐ Traumatic brain injury
- ☐ Parkinson's Disease
- ☐ Other acquired brain injury
- ☐ Neurosurgery
- ☐ Cerebral palsy
- ☐ Multiple sclerosis
- ☐ Spinal cord injury
- ☐ Vestibular
- ☐ General falls & balance
- ☐ Other – please list……………………………………..

What proportion of your neurological patients do you estimate have a problem with their walking (to the nearest 5%) ………………..

What proportion of your time spent with each neurological patient would be allocated to treating gait and gait disorders (to the nearest 5%) ………………..

What proportion of your neurological patients have a problem with muscle weakness (to the nearest 5%) ………………..

In relation to your patients with neurological gait disorders, what physical impairments do you most commonly assess (tick all that apply)?

- ☐ Joint ROM
- ☐ Muscle weakness
- ☐ Balance
- ☐ Muscle tone/spasticity
- ☐ Quality of movement (i.e. motor control/co-ordination)
- ☐ Musculoskeletal conditions
- ☐ Pain
- ☐ Other (please list)……………………………………………………………

Questions about your knowledge of clinical practice

Do you feel that you have a good understanding of the biomechanics of walking?

☐ Strongly agree

☐ Agree

☐ Neither agree nor disagree

☐ Disagree

☐ Strongly disagree

Do you feel that you have a good understanding of muscle function during walking?

☐ Strongly agree

☐ Agree

☐ Neither agree nor disagree

☐ Disagree

☐ Strongly disagree

Do you feel that you have a good understanding of the relative contributions of physical impairments to walking limitations?

☐ Strongly agree

☐ Agree

☐ Neither agree nor disagree

☐ Disagree

☐ Strongly disagree

Do you feel that you have a good understanding of the principles of training to improve muscle strength and power generation?

☐ Strongly agree

☐ Agree

☐ Neither agree nor disagree

☐ Disagree

☐ Strongly disagree

Questions about gait & gait training

How commonly (i.e. 1-7 with ‘1’ being the most common and ‘7’ being the least common) do you find the following **physical impairments limit walking** ability in the neurological patients you treat?

……. Reduced ROM

……. Reduced balance

……. Reduced cardiovascular fitness

……. Muscle weakness

……. Abnormal muscle tone

……. Poor quality of movement

……. Pain

Please rate in order (i.e. 1-7 with ‘1’ being the most time and ‘7’ being the least time) the **amount of treatment time** spent on the following physical impairments that contribute to walking limitations.

……. Reduced ROM (i.e. stretching/casting/splinting)

……. Reduced balance (i.e. balance training)

……. Reduced cardiovascular fitness

……. Muscle weakness (i.e. strength training)

……. Abnormal muscle tone (i.e. spasticity/hypertonicity/dystonia)

……. Poor quality of movement (i.e. motor control/co-ordination)

……. Pain

Which feature of the Upper Motor Neurone Syndrome (UMNS) is the most disabling in relation to gait disorders (pick one answer only)?

☐ Muscle spasticity

☐ Muscle hypertonicity

☐ Muscle weakness

☐ Reduced motor control

☐ Reduced tendon reflexes

What proportion of time would you typically devote to the following interventions for a person who has sustained a stroke and is experiencing difficulty walking (must add to 100%)

……. Stretching/casting/splinting

……. Balance training

……. Cardiovascular fitness

……. Strength training

……. Normalizing muscle tone/spasticity

……. Motor control/co-ordination

……. Pain management

……. Gait training

Considering only the time you spend on strength training with your patients with walking problems, what proportion of time do you spend on the following types of strength training (must add to 100%)?

……. General active through range movements

……. Progressive resistance training

……. Fast/ballistic training

……. Co-contraction to improve joint control and stability

……. Lighter control exercises to improve the quality of movement

Please rank in order the outcome measures you most commonly use to measure walking capacity in your patients (i.e. 1-6 with '1' being the most common and '5' being the least common)?

☐ Timed up and go

☐ 10m walk – self-selected/natural speed

☐ 10m walk – fast

☐ 6 min walk

☐ Motor FIM

☐ Other (please list)……………………………………………………….

Which of the following recommendations from the National Stroke Foundation guidelines for the management of stroke is true (tick all that apply)

- ☐ As much repetitive practice of walking as possible is not recommended
- ☐ Progressive resistance exercises should be used in the presence of weakness
- ☐ Interventions to reduce mild-moderate spasticity should be routinely provided
- ☐ Splints and prolonged stretching should be routinely provided for those at risk of developing a contracture

Questions about gait and the biomechanics of gait

Please tick the three most important muscle groups for forward propulsion when walking

- ☐ Hip extensors
- ☐ Hip abductors
- ☐ Hip flexors
- ☐ Knee extensors (i.e. quadriceps)
- ☐ Knee flexors (i.e. hamstrings)
- ☐ Plantarflexors
- ☐ Dorsiflexors
- ☐ Ankle evertors

During walking the quadriceps primarily (pick one answer only)

- ☐ Push off during stance phase
- ☐ Extend the knee in late swing phase
- ☐ Generate muscle power
- ☐ Absorb muscle power

During walking the hamstrings primarily act to (pick one answer only)

- ☐ Flex the knee during swing phase
- ☐ Control the knee during loading response
- ☐ Flex the knee in late stance phase
- ☐ Slow the leg in late swing

The hip extensors primarily act (pick one answer only)

- ☐ Throughout stance phase
- ☐ At initial contact
- ☐ During mid-stance
- ☐ In late stance

In order to walk faster, healthy able-bodied people usually (pick one answer only)

- ☐ Increase their stride length
- ☐ Increase their cadence
- ☐ Increase both equally
- ☐ Maintain their stride length and cadence but work harder

What proportion of the overall power generation for walking does the calf muscle perform? (pick one answer only)

- ☐ 20%
- ☐ 40%
- ☐ 60%
- ☐ 80%

Considering all the power generated at the ankle joint, what proportion of the power comes from the calf muscle and what proportion comes from the elastic component of the Achilles tendon

- ☐ 20% calf muscle / 80% Achilles tendon
- ☐ 40% calf muscle / 60% Achilles tendon
- ☐ 60% calf muscle / 40% Achilles tendon
- ☐ 80% calf muscle / 20% Achilles tendon

During hemiparetic gait, the main muscle groups most commonly involved in compensatory strategies to maintain forward propulsion on the affected side are (pick all that apply)

- ☐ Hip flexors
- ☐ Hip extensors
- ☐ Hip abductors
- ☐ Knee extensors
- ☐ Knee flexors
- ☐ Plantarflexors
- ☐ Dorsiflexors

Considering the entire gait cycle during normal walking, overall the (tick all that apply)

- ☐ Hip flexors generate power
- ☐ Hip flexors absorb power
- ☐ Hip extensors generate power
- ☐ Hip extensors absorb power
- ☐ Knee flexors generate power
- ☐ Knee flexors absorb power
- ☐ Knee extensors generate power
- ☐ Knee extensors absorb power
- ☐ Ankle plantarflexors generate power
- ☐ Ankle plantarflexors absorb power

The ankle plantarflexors primarily act (pick only one answer)

- ☐ During push-off
- ☐ During all of stance phase
- ☐ During early stance/loading response phase
- ☐ During mid-stance

Questions about muscle weakness & strength training

In relation to resistance/strength training, the American College of Sports Medicine (ACSM) guidelines for **specificity** may refer to (**select the incorrect answer**)

- ☐ Muscle action
- ☐ Speed of movement
- ☐ Load
- ☐ Active range and segmental alignment
- ☐ Energy systems involved

Strategies for progression when strength training include (**select the incorrect answer**)

- ☐ Progressive muscle overload
- ☐ Increasing exercise intensity
- ☐ Increasing total repetitions
- ☐ Reducing the speed of the exercise
- ☐ Increasing total training volumes
- ☐ Shortening rest periods

In relation to measurement, which of the following is correct (pick only one answer)

- ☐ Power is a measure of maximum muscle force
- ☐ Maximum muscle force is a measure of the rate of force production
- ☐ The rate of force production is a measure of power
- ☐ Muscle power and muscle force are two different terms that mean the same thing
