# Supplementary material for: Clinical education alone is sufficient to increase resistance training exercise prescription
Source: PLoS One. 2019 Feb 27;14(2):e0212168. doi: 10.1371/journal.pone.0212168 (PMC6392279; doi:10.1371/journal.pone.0212168)
Supplement: S3 File — (PDF) [file pone.0212168.s003.pdf]

Questions about your knowledge of clinical practice

Do you feel that you have a better understanding of the biomechanics of walking?

- ☐ Strongly agree
- ☐ Agree
- ☐ Neither agree nor disagree
- ☐ Disagree
- ☐ Strongly disagree

Do you feel that you have a better understanding of muscle function during walking?

- ☐ Strongly agree
- ☐ Agree
- ☐ Neither agree nor disagree
- ☐ Disagree
- ☐ Strongly disagree

Do you feel that you have a better understanding of the relative contributions of physical impairments to walking limitations?

- ☐ Strongly agree
- ☐ Agree
- ☐ Neither agree nor disagree
- ☐ Disagree
- ☐ Strongly disagree

Do you feel that you have a better understanding of the principles of training to improve muscle strength and power generation?

- ☐ Strongly agree
- ☐ Agree
- ☐ Neither agree nor disagree
- ☐ Disagree
- ☐ Strongly disagree

Questions about gait & gait training

Which feature of the Upper Motor Neurone Syndrome (UMNS) is the most disabling in relation to gait disorders (pick one answer only)?

- ☐ Muscle spasticity
- ☐ Muscle hypertonicity
- ☐ Muscle weakness
- ☐ Reduced motor control
- ☐ Reduced tendon reflexes

Which of the following recommendations from the National Stroke Foundation guidelines for the management of stroke is true (tick all that apply)

- ☐ As much repetitive practice of walking as possible is not recommended
- ☐ Progressive resistance exercises should be used in the presence of weakness
- ☐ Interventions to reduce mild-moderate spasticity should be routinely provided
- ☐ Splints and prolonged stretching should be routinely provided for those at risk of developing a contracture

Questions about gait and the biomechanics of gait

Please tick the three most important muscle groups for forward propulsion when walking

- ☐ Hip extensors
- ☐ Hip abductors
- ☐ Hip flexors
- ☐ Knee extensors (i.e. quadriceps)
- ☐ Knee flexors (i.e. hamstrings)
- ☐ Plantarflexors
- ☐ Dorsiflexors
- ☐ Ankle evertors

During walking the quadriceps primarily (pick one answer only)

- ☐ Push off during stance phase
- ☐ Extend the knee in late swing phase
- ☐ Generate muscle power
- ☐ Absorb muscle power

During walking the hamstrings primarily act to (pick one answer only)

- ☐ Flex the knee during swing phase
- ☐ Control the knee during loading response
- ☐ Flex the knee in late stance phase
- ☐ Slow the leg in late swing

The hip extensors primarily act (pick one answer only)

- ☐ Throughout stance phase
- ☐ At initial contact
- ☐ During mid-stance
- ☐ In late stance

In order to walk faster, healthy able-bodied people usually (pick one answer only)

- ☐ Increase their stride length
- ☐ Increase their cadence
- ☐ Increase both equally
- ☐ Maintain their stride length and cadence but work harder

What proportion of the overall power generation for walking does the calf muscle perform? (pick one answer only)

- ☐ 20%
- ☐ 40%
- ☐ 60%
- ☐ 80%

Considering all the power generated at the ankle joint, what proportion of the power comes from the calf muscle and what proportion comes from the elastic component of the Achilles tendon

- ☐ 20% calf muscle / 80% Achilles tendon
- ☐ 40% calf muscle / 60% Achilles tendon
- ☐ 60% calf muscle / 40% Achilles tendon
- ☐ 80% calf muscle / 20% Achilles tendon

During hemiparetic gait, the main muscle groups most commonly involved in compensatory strategies to maintain forward propulsion on the affected side are (pick all that apply)

- ☐ Hip flexors
- ☐ Hip extensors
- ☐ Hip abductors
- ☐ Knee extensors
- ☐ Knee flexors
- ☐ Plantarflexors
- ☐ Dorsiflexors

Considering the entire gait cycle during normal walking, overall the (tick all that apply)

- ☐ Hip flexors generate power
- ☐ Hip flexors absorb power
- ☐ Hip extensors generate power
- ☐ Hip extensors absorb power
- ☐ Knee flexors generate power
- ☐ Knee flexors absorb power
- ☐ Knee extensors generate power
- ☐ Knee extensors absorb power
- ☐ Ankle plantarflexors generate power
- ☐ Ankle plantarflexors absorb power

The ankle plantarflexors primarily act (pick only one answer)

- ☐ During push-off
- ☐ During all of stance phase
- ☐ During early stance/loading response phase
- ☐ During mid-stance

Questions about muscle weakness & strength training

In relation to resistance/strength training, the American College of Sports Medicine (ACSM) guidelines for **specificity** may refer to (**select the incorrect answer**)

- ☐ Muscle action
- ☐ Speed of movement
- ☐ Load
- ☐ Active range and segmental alignment
- ☐ Energy systems involved

Strategies for progression when strength training include (**select the incorrect answer**)

- ☐ Progressive muscle overload
- ☐ Increasing exercise intensity
- ☐ Increasing total repetitions
- ☐ Reducing the speed of the exercise
- ☐ Increasing total training volumes
- ☐ Shortening rest periods

In relation to measurement, which of the following is correct (pick only one answer)

- ☐ Power is a measure of maximum muscle force
- ☐ Maximum muscle force is a measure of the rate of force production
- ☐ The rate of force production is a measure of power
- ☐ Muscle power and muscle force are two different terms that mean the same thing

Implementation

How confident do you feel that you could implement a training program that is targeted towards the three main muscle groups that generate power for walking in your patients?

☐ Very confident

☐ Confident

☐ Neither confident nor

☐ Unconfident

☐ Very unconfident

What do you think are the barriers to targeted power training for your patients (please tick all that apply)?

☐ My confidence and capacity to provide power training to patients

☐ Patient capacity to engage in power generation training

☐ Time (i.e. too time consuming)

☐ Equipment (i.e. don't have access to required equipment)

☐ I don't believe it will work

☐ It's not part of my job/role to perform these exercises

☐ It's easier to stick with the exercises I am currently providing

☐ My current exercise programs are sufficient

☐ My organisation does not provide adequate support to engage in new evidence-based models of care

☐ Lacking in other resources (please specify)……………………………
