# Supplementary material for: Clinical education alone is sufficient to increase resistance training exercise prescription
Source: PLoS One. 2019 Feb 27;14(2):e0212168. doi: 10.1371/journal.pone.0212168 (PMC6392279; doi:10.1371/journal.pone.0212168)
Supplement: S4 File — (PDF) [file pone.0212168.s004.pdf]

Questions about you

This section is deliberately repetitive so we can compare the demographics of those who have responded.

Professional background:
- ☐ Physiotherapist
- ☐ Exercise Physiologist
- ☐ Exercise Scientist

Years of experience since graduation:
- ☐ < 2 years
- ☐ 3-5 years
- ☐ 6-10 years
- ☐ >10 years

Normal work hours:
- ☐ < 10 hours/week
- ☐ 10-25 hours/week
- ☐ 25-40 hours/week
- ☐ >40 hours/week

Main work setting (tick all that apply):

☐ Inpatients	☐ Outpatients	☐ Community
☐ Acute	☐ Rehabilitation	☐ Private practice
☐ City	☐ Major regional facility	☐ Rural/remote
☐ Public Hospital	☐ Private Hospital	

☐ Other………………………………………………….

Have you attended a post-graduate educational lecture/workshop/training session on the management of gait disorders for people with neurological conditions in the past 5 years?

- ☐ Yes
- ☐ No

In relation to gait disorders and gait training, where do you most commonly seek up to date knowledge and information? Please order these from 1-8 (1 – most common, 8 – least common)

- ☐ International guidelines
- ☐ Professional development activity (internal – i.e. provided by your employer)
- ☐ Professional development activity (external)
- ☐ Workshops/Conferences/seminars
- ☐ Books or Journal articles
- ☐ Online Resources
- ☐ Unit of university course
- ☐ Other (please list)……………………………………………………….

Questions about gait & gait training

How commonly (i.e. 1-7 with ‘1’ being the most common and ‘7’ being the least common) do you find the following **physical impairments limit walking** ability in the neurological patients you treat?

……. Reduced ROM

……. Reduced balance

……. Reduced cardiovascular fitness

……. Muscle weakness

……. Abnormal muscle tone

……. Poor quality of movement

……. Pain

Please rate in order (i.e. 1-7 with ‘1’ being the most time and ‘7’ being the least time) the **amount of treatment time** spent on the following physical impairments that contribute to walking limitations.

……. Reduced ROM (i.e. stretching/casting/splinting)

……. Reduced balance (i.e. balance training)

……. Reduced cardiovascular fitness

……. Muscle weakness (i.e. strength training)

……. Abnormal muscle tone (i.e. spasticity/hypertonicity/dystonia)

……. Poor quality of movement (i.e. motor control/co-ordination)

……. Pain

Which feature of the Upper Motor Neurone Syndrome (UMNS) is the most disabling in relation to gait disorders (pick one answer only)?

☐ Muscle spasticity

☐ Muscle hypertonicity

☐ Muscle weakness

☐ Reduced motor control

☐ Reduced tendon reflexes

What proportion of time would you typically devote to the following interventions for a person who has sustained a stroke and is experiencing difficulty walking (must add to 100%)

……. Stretching/casting/splinting

……. Balance training

……. Cardiovascular fitness

……. Strength training

……. Normalizing muscle tone/spasticity

……. Motor control/co-ordination

……. Pain management

……. Gait training

Considering only the time you spend on strength training with your patients with walking problems, what proportion of time do you spend on the following types of strength training (must add to 100%)?

……. General active through range movements

……. Progressive resistance training

……. Fast/ballistic training

……. Co-contraction to improve joint control and stability

……. Lighter control exercises to improve the quality of movement

Please rank in order the outcome measures you most commonly use to measure walking capacity in your patients (i.e. 1-6 with ‘1’ being the most common and ‘5’ being the least common)?

☐ Timed up and go

☐ 10m walk – self-selected/natural speed

☐ 10m walk – fast

☐ 6 min walk

☐ Motor FIM

☐ Other (please list)………………………………………………………

Which of the following recommendations from the National Stroke Foundation guidelines for the management of stroke is true (tick all that apply)

- ☐ As much repetitive practice of walking as possible is not recommended
- ☐ Progressive resistance exercises should be used in the presence of weakness
- ☐ Interventions to reduce mild-moderate spasticity should be routinely provided
- ☐ Splints and prolonged stretching should be routinely provided for those at risk of developing a contracture

Questions about gait and the biomechanics of gait

Please tick the three most important muscle groups for forward propulsion when walking

- ☐ Hip extensors
- ☐ Hip abductors
- ☐ Hip flexors
- ☐ Knee extensors (i.e. quadriceps)
- ☐ Knee flexors (i.e. hamstrings)
- ☐ Plantarflexors
- ☐ Dorsiflexors
- ☐ Ankle evertors

During walking the quadriceps primarily (pick one answer only)

- ☐ Push off during stance phase
- ☐ Extend the knee in late swing phase
- ☐ Generate muscle power
- ☐ Absorb muscle power

During walking the hamstrings primarily act to (pick one answer only)

- ☐ Flex the knee during swing phase
- ☐ Control the knee during loading response
- ☐ Flex the knee in late stance phase
- ☐ Slow the leg in late swing

The hip extensors primarily act (pick one answer only)

- ☐ Throughout stance phase
- ☐ At initial contact
- ☐ During mid-stance
- ☐ In late stance

In order to walk faster, healthy able-bodied people usually (pick one answer only)

- ☐ Increase their stride length
- ☐ Increase their cadence
- ☐ Increase both equally
- ☐ Maintain their stride length and cadence but work harder

What proportion of the overall power generation for walking does the calf muscle perform? (pick one answer only)

- ☐ 20%
- ☐ 40%
- ☐ 60%
- ☐ 80%

Considering all the power generated at the ankle joint, what proportion of the power comes from the calf muscle and what proportion comes from the elastic component of the Achilles tendon

- ☐ 20% calf muscle / 80% Achilles tendon
- ☐ 40% calf muscle / 60% Achilles tendon
- ☐ 60% calf muscle / 40% Achilles tendon
- ☐ 80% calf muscle / 20% Achilles tendon

During hemiparetic gait, the main muscle groups most commonly involved in compensatory strategies to maintain forward propulsion on the affected side are (pick all that apply)

- ☐ Hip flexors
- ☐ Hip extensors
- ☐ Hip abductors
- ☐ Knee extensors
- ☐ Knee flexors
- ☐ Plantarflexors
- ☐ Dorsiflexors

Considering the entire gait cycle during normal walking, overall the (tick all that apply)

- ☐ Hip flexors generate power
- ☐ Hip flexors absorb power
- ☐ Hip extensors generate power
- ☐ Hip extensors absorb power
- ☐ Knee flexors generate power
- ☐ Knee flexors absorb power
- ☐ Knee extensors generate power
- ☐ Knee extensors absorb power
- ☐ Ankle plantarflexors generate power
- ☐ Ankle plantarflexors absorb power

The ankle plantarflexors primarily act (pick only one answer)

- ☐ During push-off
- ☐ During all of stance phase
- ☐ During early stance/loading response phase
- ☐ During mid-stance

Questions about muscle weakness & strength training

In relation to resistance/strength training, the American College of Sports Medicine (ACSM) guidelines for **specificity** may refer to (**select the incorrect answer**)

- ☐ Muscle action
- ☐ Speed of movement
- ☐ Load
- ☐ Active range and segmental alignment
- ☐ Energy systems involved

Strategies for progression when strength training include (**select the incorrect answer**)

- ☐ Progressive muscle overload
- ☐ Increasing exercise intensity
- ☐ Increasing total repetitions
- ☐ Reducing the speed of the exercise
- ☐ Increasing total training volumes
- ☐ Shortening rest periods

In relation to measurement, which of the following is correct (pick only one answer)

- ☐ Power is a measure of maximum muscle force
- ☐ Maximum muscle force is a measure of the rate of force production
- ☐ The rate of force production is a measure of power
- ☐ Muscle power and muscle force are two different terms that mean the same thing

Implementation

Over the past 3 months, do you feel that you have increased the proportion of time targeted towards the treatment of muscle weakness for your patients?

- ☐ Strongly agree
- ☐ Agree
- ☐ Neither agree nor disagree
- ☐ Disagree
- ☐ Strongly disagree

Over the past 3 months, do you feel that you have increased the proportion of time spent on fast or ballistic strength training for your patients?

- ☐ Strongly agree
- ☐ Agree
- ☐ Neither agree nor disagree
- ☐ Disagree
- ☐ Strongly disagree

Over the past 3 months, do you feel that you have focused your strength training towards the main muscle groups responsible for forward propulsion during walking for your patients?

- ☐ Strongly agree
- ☐ Agree
- ☐ Neither agree nor disagree
- ☐ Disagree
- ☐ Strongly disagree

Over the past 3 months, have you noticed a change in the exercise prescription of your colleagues for patients with walking problems?

- ☐ Strongly agree
- ☐ Agree
- ☐ Neither agree nor disagree
- ☐ Disagree
- ☐ Strongly disagree

After the education and training session 3 months ago, we asked you about potential barriers to targeting specific and ballistic power training for walking with your patients.

Considering your exercise prescription over the past 3 months, what do you think were the barriers to targeted power training for your patients (please tick all that apply)?

☐ My confidence and capacity to provide power training to patients

☐ Patient capacity to engage in power generation training

☐ Time (i.e. too time consuming)

☐ Equipment (i.e. don't have access to required equipment)

☐ I don't believe it will work

☐ It's not part of my job/role to perform these exercises

☐ It's easier to stick with the exercises I am currently providing

☐ My current exercise programs are sufficient

☐ My organisation does not provide adequate support to engage in new evidence-based models of care

☐ Lacking in other resources (please specify)……………………………

What were the enablers (i.e. the things that helped you implement new exercises) to your change in exercise prescription (please tick all that apply)?

☐ Greater confidence and capacity to provide power training to patients

☐ Greater understanding of the contribution of physical impairments to gait disorders

☐ Greater understanding of the primary muscle groups responsible for forward propulsion when walking

☐ Greater understanding of strength training and application of exercise programs

☐ Access to suitable equipment that meets my patient's needs

☐ Support and encouragement of my colleagues/peers

☐ A belief that targeted ballistic strength training is the best intervention for my patients

☐ The new targeted ballistic exercises are better than my usual exercises

☐ The patients are more engaged with the new targeted ballistic exercises
