# Supplementary material for: Clinical education alone is sufficient to increase resistance training exercise prescription
Source: PLoS One. 2019 Feb 27;14(2):e0212168. doi: 10.1371/journal.pone.0212168 (PMC6392279; doi:10.1371/journal.pone.0212168)
Supplement: S5 File — (PDF) [file pone.0212168.s005.pdf]

University of Melbourne
Faculty of Medicine, Dentistry and Health Sciences
Department of Physiotherapy

Project Protocol

THE UNIVERSITY OF MELBOURNE

Project Title: Effective strength training for walking in neurological rehabilitation

Responsible Researcher: Dr Gavin Williams
Ph: 0400 019 386; Email: gavinw@unimelb.edu.au

Co-Researcher: Prof Linda Denehy
Ph: 03 8344 6428; Email: l.denehy@unimelb.edu.au

1. Project Summary

The main cause of reduced ability to walk for the majority of people with neurological conditions is muscle weakness. Clinical guidelines from the National Stroke Foundation (NSF) Clinical Guidelines for Stroke Management[1] and the American Heart Association Statement for Stroke[2] both recommend strength training for the lower limb to improve walking. Further, clinical guidelines have also been published by the American College of Sports Medicine (ACSM) for optimal strength training[3]. However, despite a large body of evidence that muscle weakness is the primary impairment causing walking limitations, and guidelines recommending strength training during rehabilitation, six systematic reviews have demonstrated that the application of the NSF guidelines has not improved walking outcomes[4-9]. Our research indicates that the gap is within the NSF guidelines. The NSF guidelines are vague and lack the 'How To' direction for the implementation of their strength training recommendations. The aim of this research project is to develop an education and training package for implementing the ACSM guidelines for strength training, and test whether it is effective in order to direct the application of the NSF guidelines.

2. Study Goals and Objectives

The overall aim of this research project is to determine the effectiveness of an education and training package in improving the provision of exercises to people who are attempting to improve their ability to walk. Specifically, the three main aims are;

- To evaluate the effectiveness of the education and training package for improving knowledge of the biomechanics of gait, and strength training exercises specific to walking
- To determine whether attendance at an education and training session results in changes in exercise prescription for people with mobility limitations
- To determine whether ongoing support and mentoring is associated with higher levels of change in exercise prescription

3. Rationale and Background

Effective strength training to improve walking following neurological injury requires the application of evidence in four different areas. 1) Weakness is the main cause of walking limitations following neurological injury such as stroke and brain injury[10,11]. Clinicians are well aware that strength training is important, and devote an appropriate proportion of their therapy time to strengthening exercises[12]. 2) The NSF Clinical Guidelines for Stroke Management recommend strength training. These guidelines are the largest body of evidence regarding the impact of muscle weakness in any adult neurological condition. These recommendations are supported by the American Heart Association Statement for Stroke which states that 'strengthening programs are an integral part of stroke rehabilitation, especially for the lower extremity'[2]. This large body of evidence, including six systematic reviews, has investigated the effectiveness of strength training to improve walking capacity for patients with a range of neurological conditions[4-9]. To summarize these systematic reviews, there is strong evidence that patients can make significant improvements to their leg strength, yet these gains have not translated into improved capacity to walk. The NSF CPG guidelines are vague and do not instruct how the strength training should be implemented to improve walking. Clinicians are following the recommendation for strength training from the NSF guidelines, but the evidence-practice gap is a deficit in the NSF guidelines which are vague and not supported by any advice or tools for application. A 'How To' guide, or education and training package, is required for clinicians to direct the application of the existing NSF guidelines.

In contrast to the NSF guidelines, 3) the ACSM strength training guidelines are very specific and state the key factors to consider when designing a strength training program. The ACSM guidelines consider factors such as the frequency, intensity, specificity and progression rules for strengthening programs, and each area is supported by a substantial body of knowledge. Specificity is a term used to describe how a muscle works, the type of contraction, the range in which it works, and how quickly it contracts. Although the ACSM guidelines were developed for healthy adults, they have been widely applied in many clinical trials for strength training in adults with neurological conditions[4-9]. However, their application has been poor, and most strengthening protocols were not designed to provide a training stimulus that would result in a change to walking capacity[6,13]. This second evidence-practice gap is the application of the ASCM guidelines to strength training programs in neurological rehabilitation. A 'How To' guide, or education and training package, is required for clinicians to effectively apply the ACSM guidelines to current strength training programs.

4) In addition to the poor application of the ACSM guidelines, the main muscle groups responsible for walking were not prioritized. There is overwhelming evidence as to the actions of the lower limb muscles during walking. Although a prerequisite level of leg strength is required in all muscle groups for walking, three muscle groups need to be particularly strong[14-18]. They are the ankle plantar-flexors, hip extensors and hip flexors. Therefore, it is reasonable to assume that these three muscle groups should be prioritized and selectively strengthened in order to improve walking, yet our systematic review found only 28% of trials included all three of these muscle group[13]. We found a strong bias towards testing and strengthening the thigh muscles, despite their relatively minor role during walking[13]. The third evidence-practice gap we have identified is that current strength training programs do not target the most important muscle groups for walking. A 'How To' guide, or

education and training package, is required for clinicians to selectively apply the existing ACSM guidelines to the main muscle groups responsible for walking.

The implementation of existing evidence and guidelines in these four areas to strength training programs in neurological rehabilitation may lead to a greater capacity for patients to walk, and requires no additional resources. The evidence-practice gaps we have identified relate to the content and application of targeted strengthening programs to improve walking outcomes for neurological patients.

4. Study Design and Methodology

This is a cluster randomised controlled trial. Participants for this project will be recruited from attendees at an educational and training session. Twelve rehabilitation facilities have been identified to participate in this project, representing the public and private sector, and metropolitan and rural areas. The overall study design is outlined in Figure 1. The 12 participating rehabilitation centres will be cluster randomised and will include four public, four private and four rural providers.

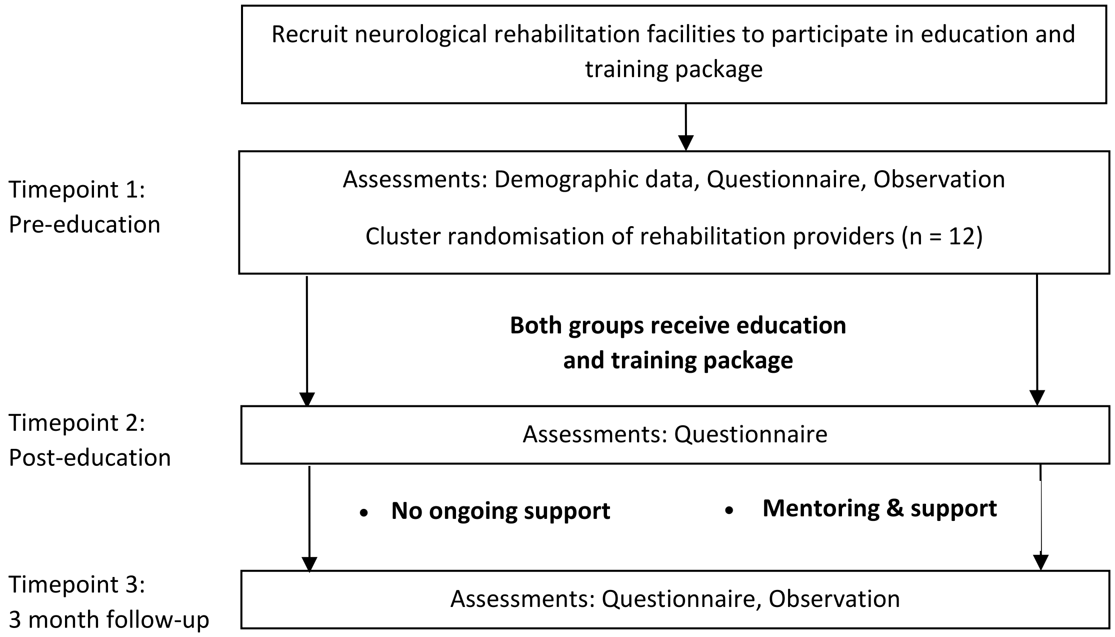

Figure 1. Study Flow Diagram

All 12 rehabilitation facilities will receive the education and training package. Six of the rehabilitation facilities will receive ongoing support and mentoring for 3 months following the education session (the intervention group), and six of the facilities will not (control group). To ensure even distribution of the different types of rehabilitation facilities in the intervention and control groups, cluster randomisation will be used (i.e. the four public facilities will be randomised, as will the private and rural providers).

***Method*:**

Twelve neurological rehabilitation facilities have been contacted and agreed to participate in this project. An education and training package, comprising a half-day workshop will be conducted at each of the participating rehabilitation facilities. All staff in the physiotherapy department will be invited to participate in the research component of the workshop prior to the workshop commencing.

Participants: The inclusion criteria are rehabilitation staff who provide informed consent and have a physiotherapy or exercise physiology qualification. Department staff who do not have one of these qualifications (for example therapy assistants or students) can attend the workshop but will not participate in the research project.

Intervention: All 12 rehabilitation facilities will be provided with a half-day workshop provided by the lead investigator. Following the workshop, half of the rehabilitation facilities will be provided with ongoing mentoring and support. The other six facilities will not.

The workshop will combine a lecture format, case presentation and practical demonstration of areas related to the biomechanics of gait, gait disorders and strength training.

The ongoing mentoring and support will be initiated by the lead investigator and will consist of mentoring boosters provided through a nominated person at the rehabilitation provider. Rehabilitation facilities randomised to the intervention group will be encouraged to raise issues related to the information in weekly team meetings, conduct their own case presentations, and support each other with peer-feedback during the 3 months following the workshop. The lead investigator will be available to respond to any questions or concerns, but the main purpose of using local peer-support and mentoring is to enhance long-term changes in exercise prescription and therapy provision.

Primary outcome measure: The primary outcome measure for this project is the amount of specific/ballistic strength training provided to improve walking outcomes. An observer will spend one day in each rehabilitation facility prior to the workshop, and a second day 3 months after the workshop. Data related to the number of neurological clients with gait disorders and what type of exercises will be recorded on the proforma attached. The number of strengthening exercises specific to walking will be recorded for comparison at each of the two observation sessions.

Secondary outcome measures: The main secondary outcome measure relates to knowledge of the biomechanics of gait, strength training exercises specific to walking, and self-report of the types of strengthening exercises used to improve walking. A questionnaire (see attached) has been developed and tested to compare baseline knowledge (time point 1), knowledge gained during the workshop (time point 2) and how knowledge has been retained (time point 3). Correct responses on the questionnaire will be used for comparison.

Data Analysis

Descriptive statistics will be used to summarise the demographic data. Independent t tests will be used to investigate demographic differences between the two groups.

A paired t test will be used to determine whether attendance at an education and training session results in changes in exercise prescription for people with mobility limitations. Observation data will be used for this analysis collected at time points 1 & 3.

Paired t tests will also be used to evaluate the effectiveness of the education and training package for improving knowledge of the biomechanics of gait, and strength training exercises specific to walking. Data from the questionnaires will be used for these analyses. Three analyses will be conducted;

1) Between time points 1 & 2 to evaluate the effectiveness of the education and training package.
2) Between time points 1 & 3 to evaluate changes in exercise prescription following the education and training package via the self-report questions
3) Between time points 2 & 3 to evaluate retention of knowledge gained from the education and training package.

An ANCOVA will be used to determine whether ongoing support and mentoring is associated with higher levels of change in exercise prescription. Observation data will be used for this analysis collected at time points 1 & 3.

5. Data Management

All data from the hardcopy questionnaires will be coded and entered into an electronic database. All hardcopies will be stored in a locked filing cabinet in the physiotherapy department office, and electronic data will be stored on a password protected computer. All participant information will remain strictly confidential. Any publications that arise from this study will only report group data, and no information that can identify any individual will be included. The data will only be available to the researchers involved in this project. The data will only be available to the lead investigators. All data will be destroyed after 5 years, in accordance with the National Health and Medical Research Council of Australia.

6. Duration of the Project

The total project will be conducted over a 2 year period.

7. Project Management

The project will be conducted by the principal researcher, Dr Gavin Williams (Physiotherapist and Research Fellow, The Department of Physiotherapy, The University of Melbourne).

8. Informed Consent Forms

Only participants who have signed a Patient Information and Consent Form (PICF) will be included in this project.

9. References

1. Foundation NS. *Clinical Guidelines for Stroke Management.* Melbourne, Australia. ISSBNO-978-0-9805933-3-4.2010.
2. Miller EL, Murray L, Richards L, et al. Comprehensive overview of nursing and interdisciplinary rehabilitation care of the stroke patient: a scientific statement from the American Heart Association. *Stroke.* 2010;41(10):2402-2448.
3. Ratamess NA, Alvar BA, Evetoch TE, et al. Progression Models in Resistance Training for Healthy Adults. *Medicine and science in sports and exercise.* 2009;41(3):687-708.
4. Kjolhede T, Vissing K, Dalgas U. Multiple sclerosis and progressive resistance training: a systematic review. *Multiple sclerosis (Houndmills, Basingstoke, England).* 2012;18(9):1215-1228.
5. van de Port IGL, Wood-Dauphinee S, Lindeman E, Kwakkel G. Effects of exercise training programs on walking competency after stroke: a systematic review. *Am J Phys Med Rehabil.* 2007;86(11):935-951.
6. Ada L, Dorsch S, Canning CG. Strengthening interventions increase strength and improve activity after stroke: a systematic review. *The Australian journal of physiotherapy.* 2006;52(4):241-248.
7. Morris SL, Dodd KJ, Morris ME. Outcomes of progressive resistance strength training following stroke: a systematic review. *Clinical Rehabilitation.* 2004;18(1):27-39.
8. Pak S, Patten C. Strengthening to promote functional recovery poststroke: an evidence-based review. *Topics in stroke rehabilitation.* 2008;15(3):177-199.
9. Dodd KJ, Taylor NF, Damiano DL. A systematic review of the effectiveness of strength-training programs for people with cerebral palsy. *Arch Phys Med Rehabil.* 2002;83(8):1157-1164.
10. Cooke EV, Tallis RC, Clark A, Pomeroy VM. Efficacy of functional strength training on restoration of lower-limb motor function early after stroke: phase I randomized controlled trial. *Neurorehabil Neural Repair.* 2010;24(1):88-96.
11. Williams GP, Schache AG, Morris ME. Mobility after traumatic brain injury: relationships with ankle joint power generation and motor skill level. *The Journal of head trauma rehabilitation.* 2013;28(5):371-378.
12. Tole G, Williams G, Clark R, Holland AE. An observational study on usual physiotherapy care in a stroke rehabilitation unit for patients with mobility deficits. *International Journal of Therapy and Rehabilitation.* 2014;21(12):585-589.
13. Williams G, Kahn M, Randall A. Strength Training for Walking in Neurologic Rehabilitation Is Not Task Specific: A Focused Review. *American Journal of Physical Medicine & Rehabilitation.* 2014;93(6):511-522.
14. Neptune RR, Sasaki K, Kautz SA. The effect of walking speed on muscle function and mechanical energetics. *Gait Posture.* 2008;28(1):135-143.
15. McGowan CP, Neptune RR, Kram R. Independent effects of weight and mass on plantar flexor activity during walking: implications for their contributions to body support and forward propulsion. *J Appl Physiol.* 2008;105(2):486-494.
16. Liu MQ, Anderson FC, Schwartz MH, Delp SL. Muscle contributions to support and progression over a range of walking speeds. *J Biomech.* 2008;41(15):3243-3252.
17. Liu MQ, Anderson FC, Pandy MG, Delp SL. Muscles that support the body also modulate forward progression during walking. *J Biomech.* 2006;39(14):2623-2630.
18. Requiao LF, Nadeau S, Milot MH, Gravel D, Bourbonnais D, Gagnon D. Quantification of level of effort at the plantarflexors and hip extensors and flexor muscles in healthy subjects walking at different cadences. *J Electromyogr Kinesiol.* 2005;15(4):393-405.
